# Do Patients with Antiphospholipid Syndrome Present with More Significant Venous Thromboembolic Clot Burden? A Retrospective Single-Center Study †

**DOI:** 10.3390/hematolrep18020021

**Published:** 2026-03-10

**Authors:** Joseph Liput, Rahim Jiwani, Rachel DiLeo, Ryan Moll, Abigail Arrigo, Yazan Samhouri, Deep Shah

**Affiliations:** 1 Allegheny Health Network Cancer Institute, Pittsburgh, PA 15212, USA; joseph.r.liput@ahn.org (J.L.);; 2 Oregon Oncology Specialists, Salem, OR 97301, USA; 3 Medicine Institute, Allegheny Health Network, Pittsburgh, PA 15224, USA; 4 Section of Hematology, Banner MD Anderson Cancer Center, Pheonix, AZ 85006, USA

**Keywords:** venous thromboembolic disease, antiphospholipid syndrome, triple positive APS

## Abstract

**Background/Objectives**: Venous thromboembolic disease (VTE) is the most common initial manifestation of antiphospholipid syndrome (APS). Determining which patients with VTE to test for APS can be a challenging clinical decision. We aimed to determine if patients with APS present with more significant venous thromboembolic clot burden, as compared to patients with VTE without a diagnosis of APS. **Methods**: A multi-hospital single-institution retrospective cohort study was designed. Patients with a diagnosis of VTE who had been tested for APS from 1 December 2019 to 31 January 2022 were included. Patients were stratified based on the presence of APS (APS versus non-APS). Significant venous thromboembolic clot burden was defined as PE involving the main and/or lobar pulmonary arteries or DVT involving the iliofemoral veins. Assessment of clot burden was performed by review of radiology reports of the index clotting event. **Results**: We included 748 patients with a history of VTE who had been tested for APS; 75 patients (10%) were positive for APS. Significant clot burden was present in 29 (38.7%) APS patients and 269 (40.0%) non-APS patients (OR 0.95, 95% CI 0.58–1.56; *p* = 0.85). No predictors for significant clot burden were found on multivariable analysis. Triple-positive APS (OR 0.83, 95% CI 0.16–4.21; *p* = 0.82) and primary APS (OR 0.72, 95% CI 0.15–3.45; *p* = 0.68) were not associated with more significant clot burden. **Conclusions**: This retrospective single-institution analysis suggests that patients with APS may not present with more significant venous thromboembolic clot burden than patients with VTE without APS.

## 1. Introduction

Antiphospholipid syndrome (APS) is a prothrombotic condition defined by arterial or venous thrombotic events and/or obstetrical morbidity in the presence of persistently positive antiphospholipid antibodies (aPL) or nonthrombotic manifestations in patients with persistent antiphospholipid antibodies [1]. Antiphospholipid antibodies target phospholipid-bound proteins and, through various incompletely defined mechanisms, render patients prone to recurrent venous, arterial, and/or microvascular clotting events [2].

Venous thromboembolic disease (VTE), comprising mostly deep vein thrombosis (DVT) and pulmonary embolism (PE), is the initial presenting clinical manifestation in approximately 30–40% of APS diagnoses, with DVT accounting for 31.7% and PE accounting for 9.0% of initial APS manifestations [3]. VTE is a heterogenous disease and can present with various degrees of clot burden. Lower extremity DVT can present with thrombus in the iliofemoral, femoropopliteal, and/or distal calf veins; pulmonary embolism can present with thrombus in the main pulmonary arteries, lobar branches, segmental, and/or subsegmental branches. In both DVT and PE, more proximal clot burden portends higher morbidity and mortality [4,5]. While VTE is a commonly encountered clinical presentation (~900k new cases annually in the United States), the decision regarding whom to test for APS is often challenging [6]. Current guidelines recommend that clinicians consider testing for APS in patients with unexplained/unprovoked venous or arterial thrombotic events, especially young patients, or when a patient has one or more specific adverse outcomes related to pregnancy [2]. To date, to our knowledge no data exist to suggest that patients with APS are more likely to present with more extensive venous thromboembolic clot burden as compared to patients without APS.

We aimed to assess clot burden in patients with a co-diagnosis of VTE and APS in comparison to patients with VTE without APS. The goal in this assessment was to determine whether clot burden should be used to determine if a patient with VTE should be tested for APS. We also investigated clinical and socioeconomic predictors of significant clot burden.

## 2. Methods

### 2.1. Study Design

We conducted a multi-hospital, single-institution retrospective cohort study at Allegheny Health Network (AHN). The study protocol was approved by the AHN institutional review board (IRB). Data were extracted from electronic medical records from 1 December 2019 to 31 January 2022. Adult patients were eligible if they had been tested for APS (APS panel, lupus anticoagulant, β2-glycoprotein antibodies, anticardiolipin antibodies) and had a known diagnosis of VTE. Patients were excluded from study participation if they had chronic VTE, upper extremity DVT, or other provoking risk factors for VTE, which included active COVID infection (within 30 days of positive test) [7], confirmed high-risk thrombophilia (Factor V Leiden homozygous, prothrombin gene mutation homozygous, compound heterozygosity, antithrombin III deficiency, protein C deficiency, protein S deficiency), active malignancy, pregnancy up to 6 weeks post-partum, and device-associated VTE. Patients were also excluded from study participation if no imaging was available for review.

### 2.2. Definitions

Patients were considered to be positive for APS if they had persistently positive antiphospholipid antibodies (aPL) and/or lupus anticoagulant (LAC) in accordance with the revised Sapporo APS Classification Criteria [8]. LAC testing included hexagonal phospholipid neutralization assay and dilute Russell viper venom time (DRVVT), with reflex. Antiphospholipid antibodies included anticardiolipin (aCL) IgG and/or IgM, which must have measured >40, and anti-β2 glycoprotein I (β2GP) antibody IgG and/or IgM in titer >99th percentile. The same test must have been positive on two or more occasions at least 12 weeks apart. Triple-positive APS was defined as persistently positive LA, aCL, and β2GP. APS was defined as secondary if the patient had a documented history of systemic lupus erythematosus and was otherwise considered primary APS. Significant clot burden was defined as PE involving the main and/or lobar pulmonary arteries or DVT involving the iliofemoral veins [9].

### 2.3. Data Collection

Diagnostic codes were utilized to identify all patients with a documented history of DVT and/or PE within the electronic medical record. Filters were then applied to only include patients who had been tested for APS (APS panel, lupus anticoagulant, β2-glycoprotein antibodies, anticardiolipin antibodies) from 1 December 2019 to 31 January 2022. Manual review of all medical records was then performed to ensure eligibility based on predefined inclusion and exclusion criteria. Data collected included age at time of VTE diagnosis, sex, BMI, race, insurance coverage, location of PE, location of DVT, APS testing results, and presence of secondary APS.

Each patient must have had a reviewable radiology report to be included in the final analysis. Computed tomography pulmonary angiography (CTPA) reports were reviewed to determine location of PE. Potential PE locations included subsegmental, segmental, lobar, and/or main pulmonary artery (including saddle) emboli. Lobar and main pulmonary artery emboli were defined as “central” emboli and were considered to represent significant clot burden. VQ scans could not be used to define extent of PE.

Lower extremity venous Doppler ultrasound and/or CT venogram reports were reviewed to determine location of DVT. In cases where discrepancies existed between lower extremity venous Doppler ultrasound and CT venogram reports, the report from the CT venogram was utilized. Potential DVT locations included iliofemoral (iliac and/or common femoral vein), femoropopliteal (superficial femoral and/or popliteal vein), and distal (gastrocnemius, peroneal, and/or tibial vein). Iliofemoral DVT was considered to represent significant clot burden.

In the case that a patient had one positive result for APS, but was not retested, they were considered to be negative for APS. In the case that a patient had one positive result and had a history of prior positive results documented in provider notes, the patient was considered to be positive for APS. In the case that patient had a history of recurrent VTE with multiple radiographic reports, imaging at time of APS testing was utilized.

### 2.4. Statistical Analysis

Patients were categorized into two groups: those with a significant clot burden and those without, as defined above. Descriptive statistics were used to compare baseline characteristics of the two groups. Continuous variables were presented as mean with standard deviation or median with interquartile range (IQR). Categorical variables were presented as absolute numbers and percentages. The means of continuous variables were compared using *t*-test, and percentages were compared using Pearson chi-square test. Univariate and multivariate logistic regression models were used to analyze predictors of clot burden. All statistical analyses were analyzed using IBM SPSS statistics v23.

## 3. Results

### 3.1. Patient Characteristics

**Figure 1:**
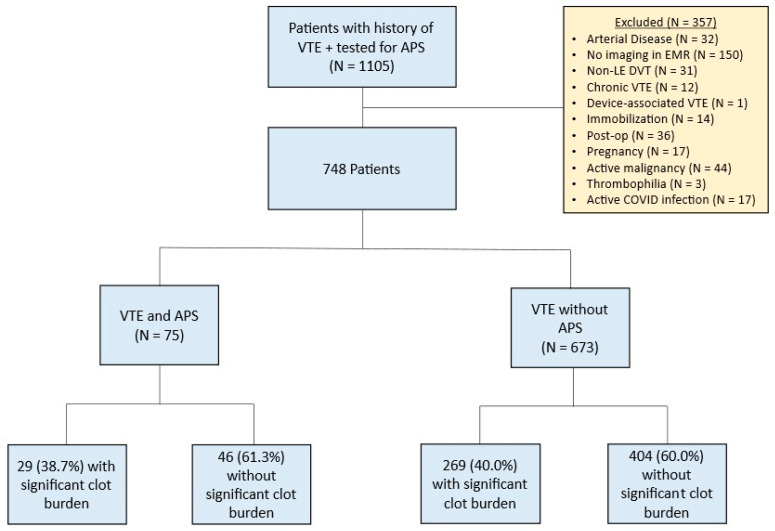
Flow chart of patients included in this study. VTE, venous thromboembolic disease; APS, antiphospholipid syndrome; EMR, electronic medical record.

The study cohort included 748 patients with a history of VTE who had also been tested for APS (Figure 1). Patient characteristics are shown in Table 1. The study population comprised 53.1% females with a median age of 59 (IQR 46–69) (Table 1). A majority of the patients were Caucasian (85%).

### 3.2. Clot Burden

Of the 748 patients included in the final analysis, 75 patients (10%) tested positive for APS. Significant clot burden was present in 29 (38.7%) APS patients and 269 (40.0%) non-APS patients (OR 0.95, 95% CI 0.58–1.56; *p* = 0.85). Of the 29 APS patients with significant clot burden, 16 had both DVT and PE (55.2%), 4 had DVT without PE (13.8%), and 9 had PE without DVT (31.0%). Of the 269 non-APS patients with significant clot burden, 128 had both DVT and PE (47.6%), 60 had DVT without PE (22.3%), and 81 had PE without DVT (30.1%).

On univariate analysis, increasing age (OR 1.01, 95% CI 1.00–1.02; *p* = 0.02) and being uninsured (OR 9.2, 95% CI 1.10–76.10; *p* = 0.04) were associated with significant clot burden. No predictors for significant clot burden were found on multivariable analysis. Triple-positive APS (OR 0.83, 95% CI 0.16–4.21; *p* = 0.82) and primary APS (OR 0.72, 95% CI 0.15–3.45; *p* = 0.68) were not associated with significant clot burden (Table 2).

## 4. Discussion

There is ongoing controversy in the field of hematology on which patients with VTE truly need to be tested for inherited and acquired thrombophilia. The most recent guidelines for thrombophilia testing published by the American Society of Hematology in 2023 further enhanced the discourse, as they recommended testing for inherited and acquired thrombophilia in all patients with VTE provoked from a non-surgical major transient risk factor [10]. This represented a notable departure from traditional practice, as many clinicians have historically viewed these episodes as provoked and managed them with short-term anticoagulation alone. They also recommended testing for inherited and acquired thrombophilia in all patients with an unprovoked VTE and clots in unusual sites. A recent meta-analysis looking at the use of direct oral anticoagulants (DOAC) versus Vitamin K antagonists (VKA) for patients with APS concluded that DOACs were associated with a much higher risk of arterial thrombosis, especially in triple-positive APS, and trending towards significance for that finding across other subgroups, including non-triple-positive APS [11]. Therefore, it has become even more important to determine which patients with VTE (given it is the most common manifestation of APS) have APS, as there can be a direct therapeutic impact on their care. Despite this, timing on when to test patients is less clear, as APS antibodies can be falsely positive in setting of acute thrombosis and while patient is actively on anticoagulation. Hence, any predictors of which patients may benefit from early testing for APS could be useful in clinical practice.

In practice, clinicians frequently become concerned of a thrombophilic condition when patients present with significant VTE clot burden. To our knowledge, this was the first study to assess for an association between VTE clot burden and APS. The results of this study would suggest that VTE clot burden may not be considered when determining whom to test for APS.

Increased age and being uninsured were associated with more significant VTE clot burden, while triple-positive APS and primary APS were not associated with more significant clot burden. No other studies were available for comparison to identify independent predictors of VTE clot burden.

Strengths of this study include strict criteria to define significant VTE clot burden. Published data suggest that central pulmonary emboli have a worse prognosis than peripheral emboli, while iliofemoral DVT portends increased morbidity as compared to more distal DVTs [12,13]. As such, these definitions were deemed to represent “significant” clot burden in our study. Another strength is the large patient pool, which included 748 patients, 75 of whom tested positive for APS (10%), which is similar to the expected prevalence of APS in patients diagnosed with VTE [14].

The limitations of our study include its retrospective design and its inherent bias. A multivariate logistic regression model was used to adjust for baseline characteristics to strengthen the results. Additionally, this study may be biased to include more patients with significant clot burden, seeing as the clinical tendency is for increased thrombophilia testing when patients have larger clots. The number of patients with triple-positive APS was low in our study, which also limits interpretation in that subgroup. This study only looked at patients who presented with a VTE as a presenting symptom of APS, and, therefore, does not cover the other prevailing manifestations of APS, such as arterial thrombosis or nonthrombotic APS, which are highlighted well in the new classification criteria for APS, published in 2023 by the American College of Rheumatology and the European Alliance of Associations for Rheumatology [15]. In terms of clot assessment, volumetric assessment using 3D-based computed tomography methods may be a more accurate measure of clot burden, as compared to location of clot [16]. It is not known, based on this study, if patients with APS and VTE have higher clot volume.

## 5. Conclusions

In conclusion, this study suggests that APS is not associated with increased VTE clot burden, as defined by location of VTE. Results would suggest that VTE clot burden may not be considered when determining whom to test for APS. Further studies are needed to determine if APS is associated with increased VTE clot volume.

## Figures and Tables

**Table 1:** Baseline characteristics.

		Significant Clot Burden (*n* = 298)	Non-Significant Clot Burden (*n* = 450)
**Age, median (IQR)**		61 (48–70)	58 (45–67)
**APS Status**	APS	29 (38.7%)	46 (61.3%)
	Non-APS	269 (40.0%)	404 (60.0%)
**APS Type**	Primary (56)	23 (41.1%)	33 (58.9%)
	Secondary (19)	6 (31.6%)	13 (68.4%)
**APS Severity**	TP (12)	3 (25.0%)	9 (75.0%)
	NTP (63)	26 (41.3%)	37 (58.7%)
**Gender**	Female	165 (41.6%)	232 (58.4%)
	Male	133 (37.9%)	218 (62.1%)
**Insurance Status**	Uninsured (9)	7 (77.8%)	2 (22.2%)
	Insured (739)	291 (39.4%)	448 (60.6%)
**BMI, median (IQR)**		32 (28–37)	31 (27–37)

Abbreviations: APS = antiphospholipid syndrome, TP = triple-positive, NTP = non-triple-positive.

**Table 2:** Predictors of significant clot burden.

Variable	Frequency (%) or Median (IQR)	Odds Ratio (95% CI)	*p* Value
**Uninsured**	9 (1.2%)	9.2 (1.10–76.10)	0.04
**Age**	59 (46–69)	1.01 (1.00–1.02)	0.02
**Female**	397 (53.1%)	1.18 (0.88–1.58)	0.27
**BMI**	32 (28–37)	1.02 (0.99–1.04)	0.06
**APS-positive**	75 (10.0%)	0.95 (0.58–1.56)	0.85
**TP APS**	12 (16.0%)	0.83 (0.16–4.21)	0.82
**Primary APS**	56 (74.7%)	0.72 (0.15–3.45)	0.68

Abbreviations: APS = antiphospholipid syndrome, IQR = interquartile range, CI = confidence interval, TP = triple-positive.

## Data Availability

Data was accessed only by the principal investigator and co-investigators on password-protected Allegheny Health Network computers and was recorded on an encrypted Excel spreadsheet. The data supporting the findings of this study are available within the article.

## Abbreviations

The following abbreviations are used in this manuscript:

VTE	Venous thromboembolic disease
APS	Antiphospholipid syndrome
aPL	Antiphospholipid antibodies
DVT	Deep vein thrombosis
PE	Pulmonary embolism
DRVVT	dilute Russell viper venom time
aCL	Anticardiolipin
β2GP	anti-β2 glycoprotein I
CTPA	Computed tomography pulmonary angiography
IQR	Interquartile range
